# A Simple Method for Removing Foreign Bodies from the Nasal Cavities of Children

**Published:** 1897-12

**Authors:** 


### A SIMPLE METHOD FOR REMOVING FOREIGN BODIES FROM THE NASAL CAVITIES OF CHILDREN.

According to Dr. G. Bieser (*Pediatrics*, July 15) the employment of the usual methods for removing foreign bodies from the nasal cavities in struggling children and without anesthesia is attended not only with the dangers from traumatism, but also with difficulty and occasional failure. The employment of serodynamics may overcome these objections. The method advised by the author is as follows: The child is placed in the ordinary position for intubation, the assistant holding his hand snugly over the child's mouth; one end of a piece of rubber tubing is snugly inserted into the nostril opposite the one holding the foreign body, the other end is inserted into the operator's mouth; the operator then blows suddenly and vigorously into the nostril and dislodges the offending body. The simplicity, cleanliness, and efficiency of this method are apparent, the child's struggles causing no traumatism.

